# Enhanced design of pCMViR-TSC plasmid vector for sustainably high cargo gene expression in mammalian cells

**DOI:** 10.1007/s11626-024-00992-2

**Published:** 2024-11-21

**Authors:** Masakiyo Sakaguchi, Rie Kinoshita, Nahoko Tomonobu, Yoshihiko Sakaguchi, Junichiro Futami, Akira Yamauchi, Hitoshi Murata, Ken-ichi Yamamoto, Tetta Takahashi, Yuma Gohara, Toshiki Ochi, Fan Jiang, Ni Luh Gede Yoni Komalasari, Youyi Chen, I Made Winarsa Ruma, I Wayan Sumardika, Jin Zhou, Tomoko Honjo, Futoshi Kuribayashi, Kazumi Sagayama, Shinichi Toyooka, Eisaku Kondo, Yusuke Inoue

**Affiliations:** 1 Department of Cell Biology, Dentistry and Pharmaceutical Sciences, Okayama University Graduate School of Medicine, 2-5-1 Shikata-Cho, Kita-Ku, Okayama-Shi, Okayama, 700-8558 Japan; 2 Department of Microbiology, Tokushima Bunri University, Sagamihara, Tokushima, Japan; 3 Department of Interdisciplinary Science and Engineering in Health Systems, Okayama University, Okayama, Japan; 4 Department of Biochemistry, Kawasaki Medical School, Kurashiki, Okayama Japan; 5 Department of Pharmacy, Okayama University Hospital, Okayama, Japan; 6 Department of Neurology, Dentistry and Pharmaceutical Sciences, Okayama University Graduate School of Medicine, Okayama, Japan; 7 Faculty of Medicine, Udayana University, Denpasar, Bali Indonesia; 8 Department of Breast Surgery, The First Affiliated Hospital, Zhejiang University School of Medicine, Hangzhou, 310003 People’s Republic of China; 9 Medical Oncology Department of Gastrointestinal Tumors, Liaoning Cancer Hospital & Institute, Cancer Hospital of the Dalian University of Technology, Shenyang, Liaoning China; 10 Organization for Research and Innovation Strategy, Okayama University, Okayama, Japan; 11 Department of General Thoracic Surgery and Breast and Endocrinological Surgery, Dentistry and Pharmaceutical Sciences, Okayama University Graduate School of Medicine, Okayama, Japan; 12 Division of Tumor Pathology, Near InfraRed Photo-Immuno-Therapy Research Institute, Kansai Medical University, Osaka, Japan; 13 Faculty of Science and Technology, Division of Molecular Science, Gunma University, Kiryu, Gunma Japan

**Keywords:** Plasmid, Gene engineering, Cancer, Cell culture

## Abstract

The first-generation pCMViR-TSC, implemented through the promoter sandwich rule, yields 10- to 100-fold higher gene expression than the standard plasmid used with the CMV (cytomegalovirus) or CAG promoter. However, the vector’s shortcomings limit its utility to transient expression only, as it is not suitable for establishing stable transformants in mammalian cells. To overcome this weakness, we here introduce the improved plasmid vector pSAKA-4B, derived from pCMViR-TSC as a second-generation chromosome-insertable vector. This vector facilitates the linear entry of the expression unit into the TTAA site of DNA universally with transposase assistance. The vector is helpful for the indefinite expression of our target gene. The new vector system is proven here to be efficient in establishing stable transformants with a high likelihood of positive clones that exhibit significantly elevated expression levels of the delivered foreign gene. This system, alongside the first-generation vector, is therefore instrumental for diverse basic research endeavors concerning genes, proteins, cells, and animals, and potentially for clinical applications such as gene therapy.

**Supplementary Information:**

The online version contains supplementary material available at 10.1007/s11626-024-00992-2.

## Introduction

Stable transformant cells established from mammalian cells engineered to overexpress a gene of interest are beneficial for assessing gene function in cells and producing the gene product as a recombinant protein. Therefore, an overabundance of tools based on plasmid- and virus-based vectors for establishing stable transformants have been developed and used worldwide thus far. These vectors have various shortcomings, such as (1) a low appearance rate of positive clones expressing the delivered gene in an intact state, (2) frequent instances of undesired high-level expression in the generated clones, (3) unexpected gene silencing leading to gene suppression, and (4) a lack of adaptation of the expression promoter unit in the vector construct to the intended cell type. As a result, the methodology for producing stable transformant cells with positive gene expression is still evolving to develop a more efficient and rapid approach to establish desired, continually expressed gene-engineered cells.

Faced with these problems, we previously generated the pIDT-SMART (C-TSC) plasmid vector (known more succinctly as pCMViR-TSC) as the first-generation vector utilizing the promoter sandwich method we developed. This method demonstrated exceptionally high expression of the inserted cargo genes (Sakaguchi *et al*. 2014b). The plasmid vector features our original C-TSC cassette (C: CMV-RU5' promoter located upstream of the cDNA; TSC: another enhancer unit composed of triple tandem enhancers, hTERT, SV40, and CMV, located downstream of the cDNA alongside a polyadenylation signal (polyA)). Due to this configuration, the developed plasmid shows far higher efficiency than robust conventional vector systems (Chen *et al*. 2019a, 2019b; Kinoshita *et al*. 2019a, 2019b; Murata *et al*. 2023; Sumardika *et al*. 2019; Takamatsu *et al*. 2019; Tomonobu *et al*. 2020a). Although the vector excels in transient expression, its inability to support the establishment of stable expression cells arises from the disruptive insertion of an antibiotic gene expression unit, tilting the optimal balance of the promoter configuration. As a result, the developed vector system is viable only for transient expression using a plasmid or adenoviral vector (Sakaguchi *et al*. 2014b, 2017; Watanabe *et al*. 2014); Putranto *et al*. 2017), rendering it unsuitable for stable expression. Thus, we aimed to enhance the plasmid pCMViR-TSC further and to establish a methodology for generating stable transformants based on pCMViR-TSC-based with the desired high gene expression.

## Materials and methods

### Cells

HEK293T (a human embryonic kidney cell line stably expressing the SV40 large T antigen; RIKEN BioResource Center, Tsukuba, Japan), MDA-MB-231 (a human TNBC cell line; ATCC, Rockville, MD), FreeStyle™ CHO-S cells (a Chinese hamster ovary cell line; Thermo Fisher Scientific, Waltham, MA), and mouse embryonic fibroblasts (MEFs) (a kind gift from Prof. Yasuhiko Yamamoto, Kanazawa University) (Takamatsu *et al*. 2019) were all cultivated in DMEM/F-12 medium (Thermo Fisher Scientific) supplemented with 10% FBS (Thermo Fisher Scientific). Because CHO-S and MEFs are rodent cells, human cells were authorized by a short tandem repeat (STR) method, identical to HEK293T cells and MDA-MB-231 cells without cell‒cell contamination (data not shown). The cells used were all checked and confirmed to be mycoplasma-negative by Hoechst 33,342 staining under both living and fixed cell conditions (data not shown).

### Plasmid vector constructs

**Figure 1.**
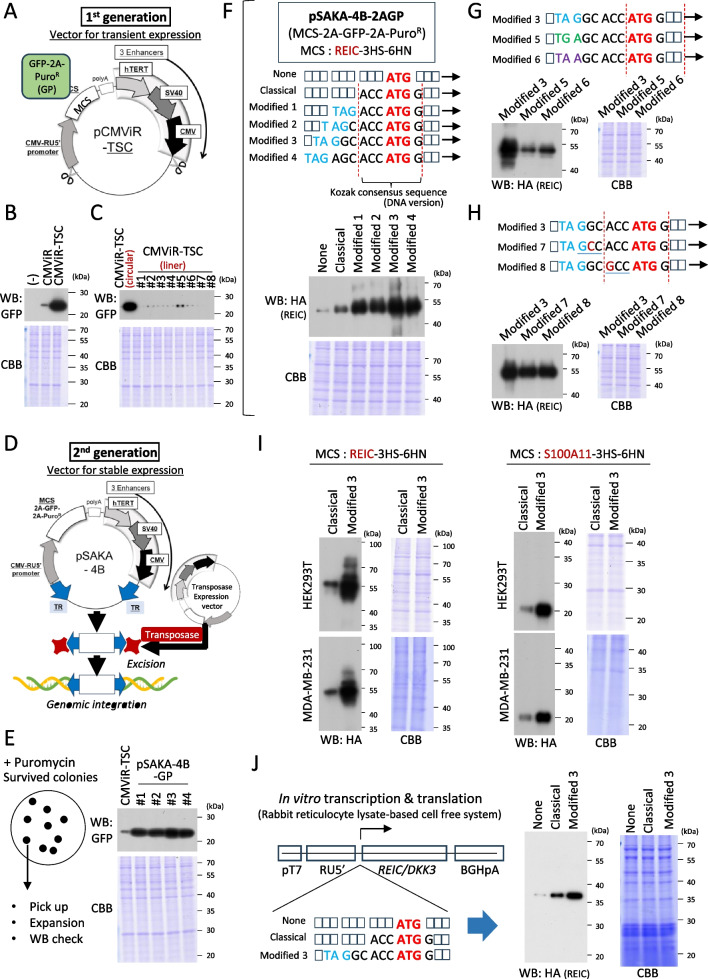
Vector arrangement. (*A, B, C*) The composition of the pCMViR-TSC (*A*) that is helpful for transient gene expression at a significant level (*B*) but not adapted to stable expression (C). GFP-2A-PuroR (GP) was placed in the MCS (*A*) to monitor GFP expression transiently (24 h) (*B*) or stably (treatment with puromycin for 3 wk) using HEK293T cells (*C*). (*D*, *E*) The second-generation plasmid vector pSAKA-4B (D) is beneficial for establishing a stable transformant of gene expression at a relatively high level in HEK293T cells (*E*). The difference in the vector composition from pCMViR-TSC lies in the leveraging of the transposon-specific inverted terminal repeat sequences (TRs) at both ends of the gene expression unit (CMV-RU5′ promoter-target gene (in MCS)-polyA-composite enhancer). Taking advantage of the TRs allows the efficient chromosomal insertion of the expression unit (CMV-RU5′-cargo gene-polyA-composite enhancer) in linear form at the TTAA site with the assistance of transposase expression from the transient vector pCMViR-TSC. (*F*) The MCS coupled with 2A-GFP-2A-PuroR was made and then replaced with the simple MCS of pSAKA-4B, resulting in the creation of pSAKA-4B-2AGP. The stop codon null REIC cDNA was then inserted into the MSC site to express a C-terminal 3 HA epitope (HA tag-HA tag-HA tag) and a 6 HN (HNHNHNHNHNHN) fusion form (REIC-3HA-6HN) (*top*). A series of modified KOZAK sequences were designed with TAG at different sites in front of the general KOZAK consensus sequence (*middle*). The designed sequences were applied to REIC-3HA-6HN cDNA, inserted into pCMViR-TSC, and delivered to HEK293T cells. The expressed REIC was detected by an anti-HA antibody (*bottom*). (*G*, *H*) The additionally designed KOZAK sequences (*upper*) were also checked for their intensity of REIC-3HA-6HN expression in HEK293T cells (*lower*). (*I*) The modified 3 version of REIC-3HA-6HN (*left*) or another S100A11-3HA-6HN (*right*) was expressed in HEK293T cells or MDA-MB-231 cells to compare with the gene expression from the control classical one (shown in *F*-*middle*). (*J*) *In* *vitro* transcription and subsequent translation reactions were done for the REIC-3HA-6HN cDNA set with none, classical, or modified 3 (shown in *F*-*middle*).

The composition of the first-generation plasmid vector, pIDT-SMART-C-TSC vector (pCMViR-TSC) (refer to Fig. 1*A* and Supplementary Fig. 1*A*), was reported previously (Sakaguchi *et al*. 2014b). The developed plasmid shows far higher efficiency than potent conventional vector systems, which are limited to transient expression. The unique structure of the plasmid is derived from adhering to the promoter sandwich rule that we uncovered. Target genes can be inserted into the multi-cloning site (MCS), where transcription is regulated mainly by the forward CMV-RU5′ promoter, composed of transcription initiation core promoter and a CMV enhancer. This regulation is remarkably enhanced by the backward composite enhancers (hTERT enhancer + SV40 enhancer + CMV enhancer). The interface between polyA and the triple enhancers has no insulator sequence. Transfection of the plasmid into cultured cells to achieve transient cargo gene expression was performed using FuGENE-HD (Promega BioSciences, San Luis Obispo, CA).

Derived from the pCMViR-TSC plasmid vector, the enhanced version was developed as a second-generation plasmid vector named pSAKA-4B (refer to Figs. 1*D* and 3*A*, and Supplementary Fig. 1*A*) to generate stable transformants based on pCMViR-TSC, which is beneficial for human cells. With two transposase (TP)-responsive terminal repeat (TR) sequences—one preceding the CMV-RU5′ and the other following the composite enhancers—the expression unit allows efficient entry into chromosomal DNA at the TTAA site, facilitated by TP obtained from co-expression with the transient expression vector pCMViR-TSC-TP. Another restructured vector, pSAKA-1B, is designed for rodent cells rather than human cells. This specificity is achieved through the incorporation of a composite sequence composed of human MARS (GenBank: Z54220.1 registered by Nikolaev *et al*. (1996)) and one human MRIRS (GenBank: AF186606.1 registered by Fullerton *et al*. (2000)), placed behind the 3′-triple tandem enhancer region (refer to Fig. 3*A*). The clones stably overexpressing our target genes were established by a convenient electroporation gene delivery method using the transient expression vector pCMViR-TSC-transposase and the Neon Transfection System (Thermo Fisher Scientific), followed by selection with puromycin at 20 µg/ml.

### Cell-free in vitro transcription and translation

A series of pCMViR-TSC-REIC/DKK-3 (REIC) plasmids, with diverse modifications to the KOZAK sequence of the REIC cDNA, are variously modified and are subjected to polymerase chain reaction (PCR) using the primer pairs (forward: 5′-CTAATACGACTCACTATAGGGAGAGCTTCGAGGGGCTCGCATCT-3′ and reverse: 3′-CCATAGAGCCCACCGCATCC-5′), which provide the T7 promoter (pT7)-RU5′-REIC-BGH pA cDNA. The products (each 100 ng) were then applied to the in vitro transcription reaction (ScriptMAX Thermo T7 transcription kit, TOYOBO, Tokyo, Japan), and the yielded mRNAs (each 1 μg) were continuously used in the in vitro translation reaction with the nuclease-treated rabbit reticulocyte lysate (Promega BioSciences).

### Cell sorting

To collect selectively the GFP-expressing cells in the stable transformant clones, the cell pellet was resuspended in 2-ml FACS buffer and passed through a 70-µm filter. GFP-positive signal was quantified using BD FACSAria III (BD Biosciences, San Jose, CA), and the data were analyzed using FlowJo software (BD Biosciences). Cell debris, doublets, and dead cells were gated out. The GFP-positive signal was identified by comparing it to the signal in non-transfected control cells. After sorting cells with a robust GFP signal, the positively sorted individual cells were collected in each well of the 96-well plate and maintained.

### Western blotting

Western blotting (WB) analysis was performed under conventional conditions, as previously reported (Gohara *et al*. 2023). The following antibodies were used: mouse anti-HA tag antibody (clone 6E2; Cell Signaling Technology, Danvers, MA), rabbit anti-GFP antibody (MBL, Tokyo, Japan), rabbit anti-human PD-L1 antibody (Gene Tex, Irvine, CA), and mouse anti-β-actin (Merck Sigma-Aldrich, St Louis, MO). The WB was conducted three times for each set of samples.

### Preparation of recombinant proteins from cell-conditioned media

The REIC protein was prepared from its corresponding conditioned medium obtained from the culture of HEK293T-derived stable cells by using the second-generation vector pSAKA-4B. After the collection of serum-free conditioned medium from a 100-mL scale culture of the established cells, these proteins were purified through Co^2+^-NTA agarose beads (Merck Sigma-Aldrich) affinity chromatography. On the other hand, the exSSSRs-Fc decoy proteins (exRAGE-Fc, exMCAM-Fc, exALCAM-Fc, exEMMPRIN-Fc, and exNPTN-Fc) were prepared from their respective conditioned media from cultures of FreeStyle™ CHO-S cell-derived stable clones. After the collection of the serum-free conditioned medium from a large-scale culture of the established CHO cell clones, the decoy recombinant proteins were purified by protein-G affinity chromatography according to the manufacturer’s instructions.

### Cell assays

We performed a Celltiter 96® Aqueous One Solution Cell Proliferation assay (an MTS assay) (Promega BioSciences) to assess cell proliferation. Cells were inoculated at a density of 2 × 10^3^ per well in 96-well plates and cultured in DMEM/F-12 medium with 0.5% FBS for 24 h. The MTS values shown were averaged from three independent experiments. In vitro cell migration activity was evaluated through a Boyden chamber assay with a transwell membrane filter insert (pore size, 8 μm) in a 24-well plate (BD Biosciences) or a wound scratch assay. In the former assay, cells (2 × 10^4^ cells/insert) were seeded in the top chamber with low-serum (0.5% FBS) DMEM/F-12 medium, while the bottom section was filled with DMEM/F12 medium containing 10% FBS. After incubation for 24 h, cells that passed through the filter were counted by staining with hematoxylin and eosin (H&E) solution. Migrated cells were imaged under a microscope (BZ-X700; KEYENCE, Tokyo, Japan) and quantified by assessing five non-overlapping fields at × 100 magnification. The number of migrated cells represents the average from three independent experiments. In the subsequent assay, 6 × 10^4^ cells were seeded into each well of the 24-well plate and grown to reach 90–100% confluence in cell density. A wound was created in the central region of the cell monolayer by scratching the plate with the edge of a 200-μL-wide pipette tip. After the scratched cell monolayer was rinsed with phosphate-buffered saline (PBS) twice, each well was filled with DMEM/F-12 medium containing 0.5% FBS. Images of the migration area were captured using an optical microscope at the 0^th^- and 12^th^-h time points.

### Statistical analysis

Each experiment was conducted three times, and the resulting raw data were statistically analyzed. The calculated values are presented as the mean ± standard deviation (SD). We used a simple pair-wise comparison with Student’s *t*-test. Values of *p* < 0.05 were considered statistically significant.

## Results

### New vector concept

Figure 1A shows a schematic overview of our established plasmid vector, pCMViR-TSC, the first-generation vector that exhibits significantly higher expression of the cargo genes compared to a single-promoter vector, CMViR, on the 5′ end (Fig. 1*B*). This enhanced expression is attributable to the developed promoter-sandwich method, in which our optimized promoters sandwich the inserted cDNA (target gene). Specifically, the CMV-RU5′ promoter is positioned at the 5′ upstream of the gene, while composite promoters composed of hTERT, SV40, and CMV are positioned at the 3′ downstream (Sakaguchi *et al*. 2014b). The utilized plasmid is helpful for transient expression but not sustainable for producing stable transformants exhibiting high-level expression of cargo genes under its circular form (data not shown because no surviving cells appeared with puromycin treatment). Even after it forms into a linear shape by cleaving it at the two designated sites, transitioning from its substantial circular form that contains GFP-2A-PuroR (GP) cDNA in the multiple cloning site (MCS), no significant GFP reporter expression was detectable in the puromycin-resistant cells (Fig. 1*C*). To enhance sustainable expression, the plasmid pCMViR-TSC was modified to create pSAKA-4B by refining the vector through iterative adjustments (the second-generation vector, Fig. 1*D*). Featuring TR sequences flanking the promoters, this construct enables the insertion of the expression unit at TTAA sites throughout the chromosomal genome. Integration typically occurs at open DNA regions where active transcription is ongoing. This chromosomal insertion method significantly boosts the ratio of positive clones expressing the delivered gene and the desired protein, as illustrated by the selection of only four colonies at random (Fig. 1*E*). In this case, the inclusion of duplicated TRs rather than a single TR could potentially enhance the efficiency of stable transformation, running in tandem with the increased expression of the inserted gene of interest. However, contrary to our expectations, having duplicated TRs at both ends of the expression unit (pSAKA-5B) led to a decreased gene expression efficiency in comparison to pSAKA-4B (Supplementary Fig. 1*A*, *B*). This unexpected result necessitates the use of a single TR for optimal configuration. The increased number of TPs bound to the duplicated TRs may disrupt the smooth process of chromosomal insertion, which includes TTAA recognition and the subsequent insertable reaction.

To assess a human gene other than the GFP reporter, we selected the REIC/DKK-3 (REIC) gene for insertion into pSAKA-4B based on our extensive research on this gene in cancer biology. Our previous studies have shown this gene to be a robust tumor suppressor with potential benefits for cancer prevention approaches (Tsuji *et al*. 2000; Tsuji *et al*. 2001; Abarzua *et al*. 2005; Tanimoto *et al*. 2010; Sakaguchi *et al*. 2011). The MCS of pSAKA-4B was slightly modified to MSC-2A-GFP-2A-PuroR, resulting in pSAKA-4B-2AGP (Fig. 1*F*, *top*), and stop-codon-negative REIC cDNA was attempted to be placed within its MSC to express REIC, GFP, and Puro^R^ simultaneously and discretely. However, before inserting the REIC cDNA into the MSC site, we explored the potential benefits of including the KOZAK consensus sequence. We have long believed intuitively that the TAG alignment could enhance cargo gene expression consistently in plasmids. This concept emerged from our cumulative observation that the restriction sites of Xba1 (TC**TAG**A), Nhe1 (GC**TAG**C), and Spe1 (AC**TAG**T) placed in front of the KOZAK consensus sequence “ACCATGG (DNA version)” (Supplementary Fig. 1*C*) consistently showed a marked increase in foreign protein levels. However, this observation is purely based on intuition in plasmid-based transient overexpression experiments in mammalian cell cultures. To clarify this, we examined in detail the significance of the TAG alignment located in the head of the KOZAK consensus sequence in detail using the REIC gene as a test gene. First, four modified sequences (modified 1–4) were created with TAG positioned at different places in front of the KOZAK consensus sequence on the REIC cDNA (Fig. 1*F*, *middle*). These constructs were compared with two control groups: one lacking the KOZAK consensus sequence (none) and the other featuring the standard KOZAK consensus sequence (classical). They were then inserted into the pCMViR-TSC plasmid vector and expressed in HEK293T cells. Notably, all the TAG-containing modified sequences exhibited higher expression levels compared to the two control constructs (Fig. 1*F*, *bottom*). In particular, the expression level caused by the modified 3 surpassed all others. Notably, a high expression comparable to that of the modified 3 was observed in the modified 4, where TAG runs as a stop codon owing to codon frame matching, which may be expected to interfere with the adjacent translational start. Owing to three variations of stop codons, another TGA and TAA were investigated for their effects on REIC expression based on the modified 3 alignment (Fig. 1*G*, *upper*), revealing no appreciable enhancement for either (Fig. 1*G*, *lower*). Because the optimized short KOZAK consensus sequence is A/GCCATGG derived from a more extended sequence, GCCA/GCCATGG (Supplementary Fig. 1*C*), we further analyzed additional alignments, modified 7 and 8 (Fig. 1*H*, *upper*). However, both sequences had almost the same expression levels as the modified 3 (Fig. 1*H*, *lower*), eventually leading to the optimal modification of the general KOZAK sequence, “TAGGCACCATGG,” as shown by the modified 3.

The optimized sequence was also applicable to different types of MDA-MB-231 breast cancer cells for the expression of either REIC (Fig. 1*I*, *left*) or a different cargo gene, S100A11 (Fig. 1*I*, *right*). A cell-free in vitro translation experiment was carried out on the modified 3 sets on the REIC cDNA to investigate the enhancement rule of the TAG-KOZAK composite. As shown in Fig. 1*J*, the modified 3 displayed the highest translation level compared to the none and classical alignments. Notably, the protein bands of REIC appeared to be smaller than those observed in the cell transfection experiments due to the lack of glycosylation modification in this cell-free system, as REIC is heavily glycosylated in the endoplasmic reticulum and the subsequent Golgi apparatus secretory pathway living cells (Kinoshita *et al*. 2015). From these results, we ultimately decided to use the modified 3 version of the KOZAK sequence for foreign gene expression in the newly developed plasmid vectors (Fig. 1*A*, *D*), ensuring that subsequent experiments involving our cDNAs of interest inserted into various vector constructs contain this specific modified 3-KOZAK sequence.

### Establishment of REIC or S100 family gene force‒expressed stable cells

**Figure 2.**
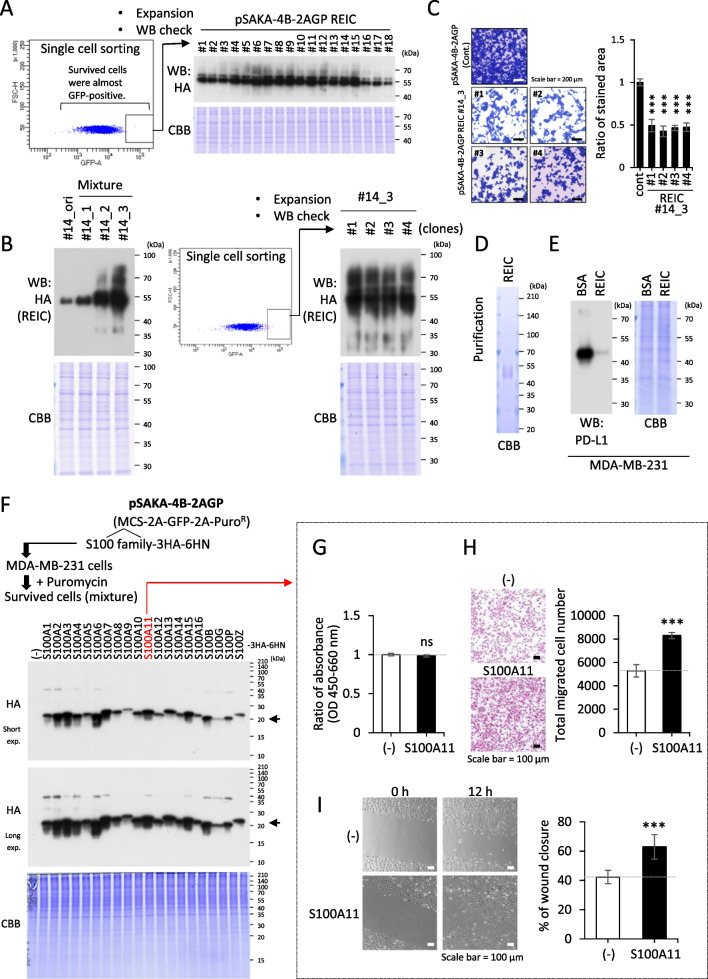
Establishment of the target gene-overexpressing cells in a stable manner with pSAKA-4B. (*A*) The newly arranged vector pSAKA-4B-2AGP-REIC, which expresses REIC-3HA-6HN, GFP, and PuroR simultaneously, was transferred into HEK293T cells, treated with puromycin, and eventually collected as a mixture of the surviving cells. The mixture was subjected to single sorting for the GFP solid-positive cells (*left*), and their parts (18 clones) were confirmed for REIC expression by the WB procedure using anti-HA-antibody (*right*). (*B*) The number 14 clone (#14), selected without bias, was then repeatedly introduced with the same vector three times via electroporation, and the transduced cells were collected as a mixture at each step (first time: #14_1, second time, #14_2, third time: #14_3). The REIC expression of the collected cells was confirmed (*leftmost*). Mixture #14_3 was subjected to single-cell sorting to target cells with solid GFP signals (*middle*). Among the sorted clones, four were chosen randomly and without bias (#14_3_#1, #2, #3, and #4), and their REIC expression was confirmed (*rightmost*). (*C*) The clones #14_3_#1, #2, #3, and #4, and control GFP stably expressed cells (2 × 10^4^ cells) were seeded into a 24-well plate (*n* = 3) and cultured for 48 h. Subsequently, all cells were stained by Coomassie brilliant blue (CBB). The BZ-X analyzer image analysis software, integrated with the BZ-X700 KEYENCE microscope, quantified the stained area. (*D*) The secreted foreign REIC protein was purified from the conditioned medium after a 72-h serum-free culture of clone #14_3_#1 using Co.^2+^-NTA agarose beads, which bind more effectively to the 6HN site compared to the 6His site of the expressed REIC. (*E*) The purified REIC protein was added to the culture of MDA-MB-231 cells at a final concentration of 50 µg/ml for 6 h. The same conc. of BSA was used as a negative control. (*F*) The pSAKA-4B-2AGP vector bearing all human S100 family genes was transfected into MDA-MB-231 cells, and the individual surviving cells were collected as a mixture (*upper*). These mixtures were all confirmed for the expression of each S100 family protein using an anti-HA antibody (*lower*). (*G*, *H*, *I*) Among the prepared mixed cell populations, the S100A11-expressing cells were studied for their cancerous abilities in growth activity (MTS assay, *G*) and migration outgrowth (trans-well assay, *H*; scratching assay, *I*).

First, REIC cDNA was inserted into the MSC of pSAKA-4B-2AGP, which enables the simultaneous expression of REIC, GFP, and Puro^R^ individually due to their linkage with the 2A sequence, facilitating efficient ribosomal skipping. Owing to the superior expression efficiency of the far-left cDNA compared to the subsequent cDNAs, the MCS was positioned on the leftmost side (Fig. 1*F*, *top*). On the other hand, Puro^R^ cDNA was positioned on the rightmost side to enable straightforward screening of the surviving cells with puromycin, anticipating robust expression of the target gene in the surviving cells due to sufficient expression of Puro^R^. In the 2A system, expression of the second gene is one-half or one-third lower than that of the first gene. Consequently, the third gene (Puro^R^) exhibits the lowest expression level. Following an electroporation procedure, the construct was introduced into HEK293T cells for 24 h accompanied by pCMViR-TSC-TP, where TP is optimized for human codons. The cells underwent to positive selection for the expression of the foreign REIC gene through puromycin treatment. The surviving cells were collected as a mixture, and each was individually sorted using fluorescence-activated cell sorting (FACS), with the objective of isolating those displaying strong GFP positivity (surrounded by squares, Fig. 2*A*, *left*). These cells were then individually placed into a 96-well plate, from which 18 clones originating from single cells were randomly and impartially selected and assessed for REIC expression across all clones in an almost uniform fashion (Fig. 2*A*, *right*).

Owing to the nature of the expression unit sandwiched between two TRs, which are insertable into the transposase-responsive TTAA site, abundant in the host DNA, the concept of enhancing cargo gene expression through repeated plasmid introductions was then examined. As expected, REIC expression gradually increased through successive electroporations conducted at regular intervals of 1 to 2 wk, following a procedure aimed at restoring cells to an optimal state with sufficient cell numbers after the cell damage induced by plasmid transduction (Fig. 2*B*, *leftmost*). The cells that had been transduced three times (#14_3) were collected and subjected to individual sorting according to the procedure described in Fig. 2*A* (Fig. 2*B*, *middle*). The four selected cells exhibited uniform patterns of high-level REIC expression (Fig. 2*B*, *rightmost*). REIC overexpression generally inhibits cell growth (Tsuji *et al*. 2001), a phenomenon observed in our established REIC-overexpressing stable cells, which consistently exhibited slower growth rates compared to the control GFP-overexpressing stable cells (Fig. 2*C*).

Our recent work has also revealed a new function of the secreted REIC protein: it functions to reduce programmed cell death ligand 1 (PD-L1), an immune checkpoint protein in cancer cells (Gohara *et al*. 2023). To test whether the pSAKA-4B‒produced REIC is functional, the REIC protein secreted into the culture medium of the #14_3 clone was purified (Fig. 2*D*) and then administered to human MDA-MB-231 breast cancer cells. This intervention reduced PD-L1 levels in the cancer cells treated with REIC, with no significant impact on the control group treated with BSA (Fig. 2*E*). Besides REIC, we have long been studying S100 genes, which are of great interest in the realms of cellular and molecular biology and pathophysiology (Sakaguchi & Huh 2011; Sakaguchi *et al*. 2014a; Ruma *et al*. 2016; Sakaguchi *et al*. 2016; Kinoshita *et al*. 2019b; Mitsui *et al*. 2019; Takamatsu *et al*. 2019; Tomonobu *et al*. 2020b).

Our next step was to establish stable cells that overexpressed the entire S100 family in MDA-MB-231 cells using the pSAKA-4B-2AGP vector. As demonstrated in the REIC expression studies (Fig. 2*A*, *B*), the vector can induce nearly all positively expressing cells with the delivered foreign gene when treated with antibiotics. In this experiment, we decided to collect the puromycin-surviving cells without resorting to colony picking or cell sorting, a strategy that facilitated the rapid generation of the targeted cell populations while mitigating potential biases during clone selection. This tactic readily led to the stable overexpression of all sorts of S100 family genes in individual cells (Fig. 2*F*). Among these genes, a particular type, S100A11, has been identified as an accelerator of cancer cell migratory outgrowth, which we have revealed (Mitsui *et al*. 2019; Takamatsu *et al*. 2019; Takahashi *et al*. 2024; Zhou *et al*. 2024). This finding is significant, as it contributes to our understanding of cancer cell behavior and could potentially lead to new therapeutic strategies. We therefore tried to confirm that the pSAKA-4B-2AGP‒mediated S100A11-overexpressing cells exhibited similar traits. Our findings indicated that while there was no significant change in proliferation (Fig. 2*G*), cell migratory activity was significantly increased. This enhancement was evident through two distinct methods: the Boyden chamber-based transwell assay (Fig. 2*H*) and the scratch-wound healing assay (Fig. 2*I*).

### Another new vector concept beneficial to rodent cells

**Figure 3.**
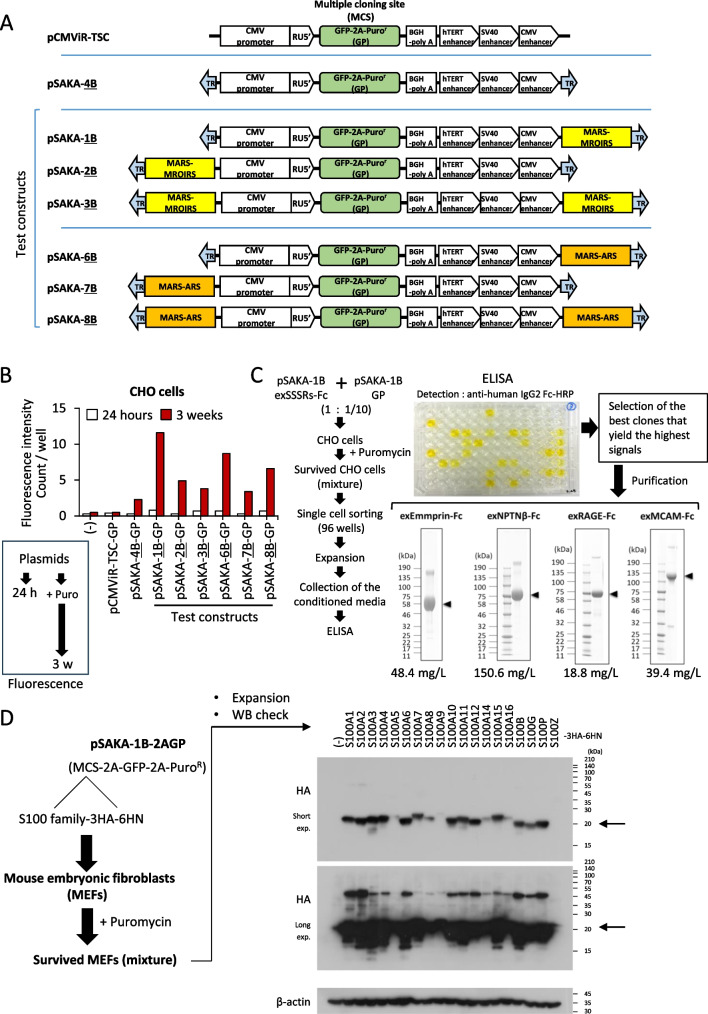
Vector arrangement applicable to rodent cells. (*A*) Schematics of the new test constructs (pSAKA-1B, 2B, 3B, 6B, 7B, and 8B) were designed and made based on the models of pCMViR-TSC and pSAKA-4B. The most significant modification is the insertion of the MARS-MROIRS composite sequence for pSAKA-1B, 2B, and 3B, and the insertion of MARS-ARS sequences for pSAKA-6B, 7B, and 8B. (*B*) These prepared vectors were all transfected into CHO cells and cultured for 24 h or 3 wk with puromycin to evaluate their GFP expression efficiencies both transiently and stably. (*C*) A pair of exSSSRs-Fc decoys were prepared from their corresponding CHO stable transformants according to a concise methodology (*left*); the ELISA image is an example of the result of the evaluation of exEMMPRIN-Fc production rates. With this approach, we successfully generated all decoy proteins displayed by CBB staining with high yields. (*D*) The pSAKA-1B-2AGP vector bearing all human S100 family genes was delivered into mouse embryonic fibroblasts (MEFs), and the surviving cells were collected as a mixture (*left*). These obtained mixtures were confirmed for the expression of each S100 family protein using an anti-HA antibody (*right*).

Based on our evolving understanding of pCMViR-TSC, pSAKA-4B, and its modified derivative pSAKA-4B-2AGP from our expression studies, these insights are somehow inapplicable to rodent-derived cells such as Chinese hamster ovary (CHO) cells, wherein gene expression levels are relatively low. Faced with this critical matter, we tried at this time to enhance the vector. Shimizu *et al*. proposed an excellent methodology involving a composite sequence composed of the matrix attachment region sequence (MARS) and the mammalian replication origin initiation region sequence (MROIRS), which proves to be highly advantageous for the stable expression of foreign genes, even in CHO cells (Araki *et al*. 2012). Upon revealing the DNA sequence, we originally selected a human MARS (GenBank: Z54220.1 registered by Nikolaev *et al*. (1996)) and a human MRIRS (GenBank: AF186606.1 registered by Fullerton *et al*. (2000)), or a similar human autonomously replicating sequence (ARS, 456 bp) (GenBank: X00300.1 registered by Montiel *et al*. (1984)). This selection led to the creation of two distinct composite sequences: MARS-MROIRS and MARS-ARS. The positional arrangement of these sequences is a critical factor influencing cargo gene expression. To evaluate this, three test constructs were employed for each MARS-MROIRS (pSAKA-1B, 2B, and 3B) or MARS-ARS (pSAKA-6B, 7B, and 8B), with their stable expression mechanisms compared to the original pCMViR-TSC and pSAKA-4B after the insertion of GP into the MCS of all constructs (Fig. 3*A*). As shown in Fig. 3*B*, both pCMViR-TSC and pSAKA-4B exhibited minimal functionality in CHO cells, whether transiently (24 h) or stably (3 wk). Conversely, the newly developed plasmids incorporating both MARS-MROIRS and MARS-ARS all worked well, particularly in stable expression with some variability. Notably, the highest expression of GFP was achieved using pSAKA-1B. Hence, we decided to use pSAKA-1B as the most applicable vector for stable expression in CHO and other rodent cell lines.

### Establishment of rodent cell‒based gene force‒expressed stable cells

Among the S100 family proteins, S100A8/A9, a heterodimer composed of S100A8 and S100A9, is a notable factor that induces inflammation-mediated cancer metastasis when present at elevated levels in the extracellular environment (Tomonobu *et al*. 2022). To implicate S100A8/A9 in life-threatening cancer symptoms, a functional aspect of S100A8/A9 as a cell surface ligand, known as the S100A8/A9 soil sensor receptor (SSSR), was necessary (Tomonobu *et al*. 2020b). Besides the classical SSSRs, toll-like receptor 4 (TLR4, gene name: TLR4) and the receptor for advanced glycation end products (RAGE, gene name: AGER), we newly identified novel SSSRs, including melanoma cell adhesion molecule (MCAM, gene name: MCAM), activated leukocyte cell adhesion molecule (ALCAM, gene name: ALCAM), extracellular matrix metalloproteinase inducer (EMMPRIN, gene name: BSG), and neuroplastin (NPTN, gene name: NPTN), as we and others have reported elsewhere (Ruma *et al*. 2016; Sakaguchi *et al*. 2016; Sumardika *et al*. 2019; Bajkowska *et al*. 2020; Herik Rodrigo *et al*. 2022). Building on these discoveries, the newly utilized S100A8/A9 inhibitor biologics, including S100A8/A9 neutralizing monoclonal antibody Ab45 and SSSR decoys (comprising recombinant fusion proteins of the extracellular domains of SSSRs (exSSSRs) linked with the human IgG2 Fc region at their C-terminal ends: exSSSRs-Fc), effectively function to prevent extracellular S100A8/A9-mediated cancer metastasis (Tomonobu *et al*. 2020a). Owing to the insufficient explanation of decoy production in a previous report (Kinoshita *et al*. 2019a), we herein explain in detail the procedure and yields of the SSSR decoy recombinant proteins in CHO cells using the pSAKA-1B vector.

Drawing from our accumulated experimental knowledge, the division of the system into pSAKA-1B exSSSRs-Fc for recombinant proteins and pSAKA-1B-GP for antibiotic expression, with a mixed ratio of about 1:1/10 between the two, has proven to be more effective than the simultaneous setup in pSAKA-1B-2AGP for enhancing recombinant protein production efficiency. According to the established protocol (Fig. 3*C*), single-cell sorting was performed to isolate the robust GFP-positive CHO cells from the puromycin-survived population. These cells were then cultured in a 96-well plate. Their conditioned media were collected and checked for exSSSRs-Fc contents using ELISA. The individual clones displaying the strongest signals were grown in culture and their large-scaled conditioned media (1L) were collected for the purification of exSSSRs-Fc. Interestingly, the purified proteins that appeared as distinct single bands on Coomassie brilliant blue (CBB) yielded large amounts, i.e., 48.4 mg, 150.6 mg, 18.8 mg, and 39.4 mg for exEMMPRIN-Fc, exNPTNβ-Fc, exRAGE-Fc, and exMCAM-Fc, respectively.

On the other hand, the vector was designed to facilitate the generation of stable gene expression transformants rather than to produce recombinant proteins for other rodent cells, such as mouse embryonic fibroblasts (MEFs). In this case, we again used S100 family genes positioned within the MCS of the pSAKA-1B-2AGP vector. Notably, this approach proved more efficient in yielding stable transformants than the separate utilization of pSAKA-1B and pSAKA-1B-GP (Fig. 3*D*, *left*). The puromycin-surviving cell mixtures were then examined for their expressions of S100 proteins, revealing strong visible expressions across all genes except for S100A13 (Fig. 3*D*, *right*), owing to the significantly slower growth rate of its transduced cell population in MEFs. Consequently, the extract from this cell population was not obtained simultaneously with the other samples in this experiment. Taking these findings together, we ultimately achieved success in harnessing beneficial plasmid vectors, namely pSAKA-4B (pSAKA-4B-2AGP) and pSAKA-1B (pSAKA-1B-2AGP), suitable for producing stable transformants with high gene expression levels in human and rodent cells, respectively. These advancements build upon the transient high gene expression vector pCMViR-TSC.

## Discussion

In this study, we developed our second-generation gene expression system that leverages the transposon-mediated plasmid genomic insertion principle. This approach enables the convenient and speedy generation of stable transformants with high-level expression of cargo genes in mammalian cells. The readout vectors are pSAKA-4B (pSAKA-4B-2A-GP) and pSAKA-1B (pSAKA-1B-2AGP), which are beneficial for human and rodent cells, respectively. It remains quite unclear why the two are not commonly applicable to different species—that is, pSAKA-4B is specifically compatible with human cells, while the MARS-MROIRS‒containing pSAKA-1B exhibits a strong affinity to rodent cells but not human cells (MARS-ARS similarly does not align well with human cells) (Supplementary Fig. 1*D*). Therefore, this challenging topic forms a part of our ongoing studies. Given the overabundance of transposase-responsive TTAA sequences within chromosomal DNA, the expression ratio correlated directly with the number of intact insertions; of course, these insertions necessitate transcriptionally active sites. Thus, iterative transduction with the vector is acceptable for further increasing the expression of the target gene.

The primary topic of discussion pertains to the positioning of an antibiotics-resistant gene, either within the gene itself or within the promoter-gene-polyA (expression unit). This aspect holds critical significance; we have been grappling with the challenge of determining the most effective positioning, as insertions of the expression unit outside the MCS disturbed the gene expression potential of pCMViR-TSC even when arranged in various configurations. Moreover, when inserted into the MCS with the help of an internal ribosomal entry site (IRES), it somehow fails to exhibit the desired expression levels, even when employing various IRES types from viral and human origins, except for the 2A sequence. This indicates that the pCMViR-TSC promoter sandwich system has poor compatibility with IRES alignments for reasons that remain unidentified (unpublished data). This experience ultimately led us to develop a strategy for segregating the expression constructs, involving a combination of the pSAKA-4B (1B)-targeted gene and the pSAKA-4B (1B)-antibiotic resistant gene in a ratio of 1:1/10. Alternatively, a more convenient approach was achieved with pSAKA-4B (1B)-2AGP, which demonstrates strong compatibility with our high-expression system, particularly due to the excellent affinity of 2A. In addition to these, we developed analogous constructs such as pSAKA-4B (1B)-2ARB, which concurrently express red fluorescence protein (RFP) and the blasticidin-resistant gene (Blas^R^). Given the distinct functional pathways of puromycin and blasticidin, Puro^R^ and Blas^R^ have a strong affinity for each other, rendering the two vectors simultaneously effective within cells in accordance with the study’s objectives.

The second point warranting discussion revolves around how the promoter sandwich elicits relatively high expression of the cargo gene, a critical question that remains to be elucidated. We previously revealed that the promoter sandwich methodology greatly enhances the transcription reaction of the interstitial cargo gene to an impressive extent, without leading to an increase in the amplification of plasmid copy numbers within the transfected cells. The heightened transcriptional activity is independent of the enhancer alignment orientation placed at the 3′-end (Sakaguchi *et al*. 2014b). Transcription extends to the end of the enhancer region. The cytosolic transposition ratio of the mRNA produced by the promoter-sandwiched vector from the nucleus is about twofold higher than that of the single non-sandwiched promoter vector. Although these events represent distinct features of the promoter-sandwiched vector, they alone do not sufficiently explain this system's incredible gene expression levels. One clue to disentangling the regulatory maze behind the high expression levels could stem from our previous experimental insights into the assembly of the promoter-sandwiched vector. The promoter sandwich rule, which leads to relatively high expression of the inserted gene, was initially uncovered during the construction and evaluation processes of the dual-gene expression vector. In this vector, two distinct promoters individually drive the expression of two other genes of interest: the 5′-CMV promoter-REIC-polyA and the hTERT promoter-JNK1-polyA-3′. The vector construct is predicated on the concept that cancer-specific overexpression of JNK1 should enhance REIC-mediated cancer-selective apoptosis. This supposition is rooted in the understanding that JNK1 plays a pivotal role in instigating cancer cell apoptosis upon the enforced expression of REIC in cancer cells. Upon constructing the vector as described above, we surprisingly found that JNK1 expression is significantly low even when changing to the same CMV promoter, while the former REIC gene exhibited remarkable expression levels. This unveiled our mistake of neglecting to include an insulator element between the two expression units to escape promoter interference. While the vector did not work correctly, the intriguing expression results led us to uncover the promoter sandwich rule in the plasmid vector. This rule correlates with relatively high gene expression of the former cargo gene situated between two promoters. Afterwards, our investigations revealed that the enhancer element, even without a transcription initiation region, can be adequately substituted with the 3′-end backward promoter. Conceivably, the recruited and gathered transcription factors on the enhancer regions of the forward promoter and the remote backward enhancer might interact in the absence of the insulator, promoting the activation of RNA polymerase. This scenario could entail modifications to the vector’s composition.

Besides the above-discussed vector arrangements, another key point involves a modification of the KOZAK consensus sequence with the TAG alignment preceding it. This modification provides higher translation activity than the standard sequence. This original finding was the culmination of researchers’ scientific intuition based on extensive experience in constructing vectors. The TAG sequence does not align in consensus with the KOZAK consensus sequence (Supplementary Fig. 1*C*), so it is entirely artificial. Nevertheless, this sequence proves beneficial for inducing protein translation at a high level. Therefore, this discovery holds promise for advancing plasmid-vector science. However, the mechanism by which the TAG sequence operates to elevate translation remains unclear. Generally, the KOZAK sequence is vital for increasing the ribosomal recognition ratio of the start codon and recruiting the initiator methionine-tRNA but not the elongator one, thereby augmenting translational initiation efficiency. The former TAG alignment may further enhance the potential of the KOZAK sequence. This enigma stands as our primary focus in upcoming investigations within this vector study.

## Conclusion

Here, we reported our second-generation vector that supersedes the first-generation vectors, facilitating the straightforward generation of stable single or mixed-cell populations with markedly high efficiency in a sustainable manner. Through the rapid establishment of target cells expressing the desired genes, this technique proves valuable for various fundamental research endeavors involving genes, proteins, and cells in both cultured systems and animals.

## Supplementary Information

Below is the link to the electronic supplementary material.

Supplementary file1 Additional data on the process of vector remodeling. A, The key distinction between pSAKA-4B and pSAKA-5B lies in the number of TRs. B, The prepared vectors (pCMViR-TSC, pSAKA-4B, and pSAKA-5B) were all delivered into HEK293T cells and cultured for 24 hours or 3 weeks with puromycin to assess their GFP expression levels both transiently and stably. C, The well-known general KOZAK consensus sequence is displayed in an image generated using WebLogo3 (https://weblogo.threeplusone.com/) based on the start codon surrounding regions of 699 vertebrate genes. D, The test constructs (pSAKA-1B, 2B, 3B, 6B, 7B, and 8B), pCMViR-TSC, and pSAKA-4B were all delivered into HEK293T cells and cultured for 24 hours or 3 weeks with puromycin to assess their GFP expression levels both transiently and stably (PPTX 276 KB)
