# Supplementary material for: Enhanced design of pCMViR-TSC plasmid vector for sustainably high cargo gene expression in mammalian cells
Source: In Vitro Cell Dev Biol Anim. 2024 Nov 21;60(10):1215–27. doi: 10.1007/s11626-024-00992-2 (PMC11655592; doi:10.1007/s11626-024-00992-2)
Supplement: Supplementary file 1 — Supplementary file1 Additional data on the process of vector remodeling. A, The key distinction between pSAKA-4B and pSAKA-5B lies in the number of TRs. B, The prepared vectors (pCMViR-TSC, pSAKA-4B, and pSAKA-5B) were all delivered into HEK293T cells and cultured for 24 hours or 3 weeks with puromycin to assess their GFP expression levels both transiently and stably. C, The well-known general KOZAK consensus sequence is displayed in an image generated using WebLogo3 (https://weblogo.threeplusone.com/) based on the start codon surrounding regions of 699 vertebrate genes. D, The test constructs (pSAKA-1B, 2B, 3B, 6B, 7B, and 8B), pCMViR-TSC, and pSAKA-4B were all delivered into HEK293T cells and cultured for 24 hours or 3 weeks with puromycin to assess their GFP expression levels both transiently and stably (PPTX 276 KB) [file 11626_2024_992_MOESM1_ESM.pptx]

## Slide 1
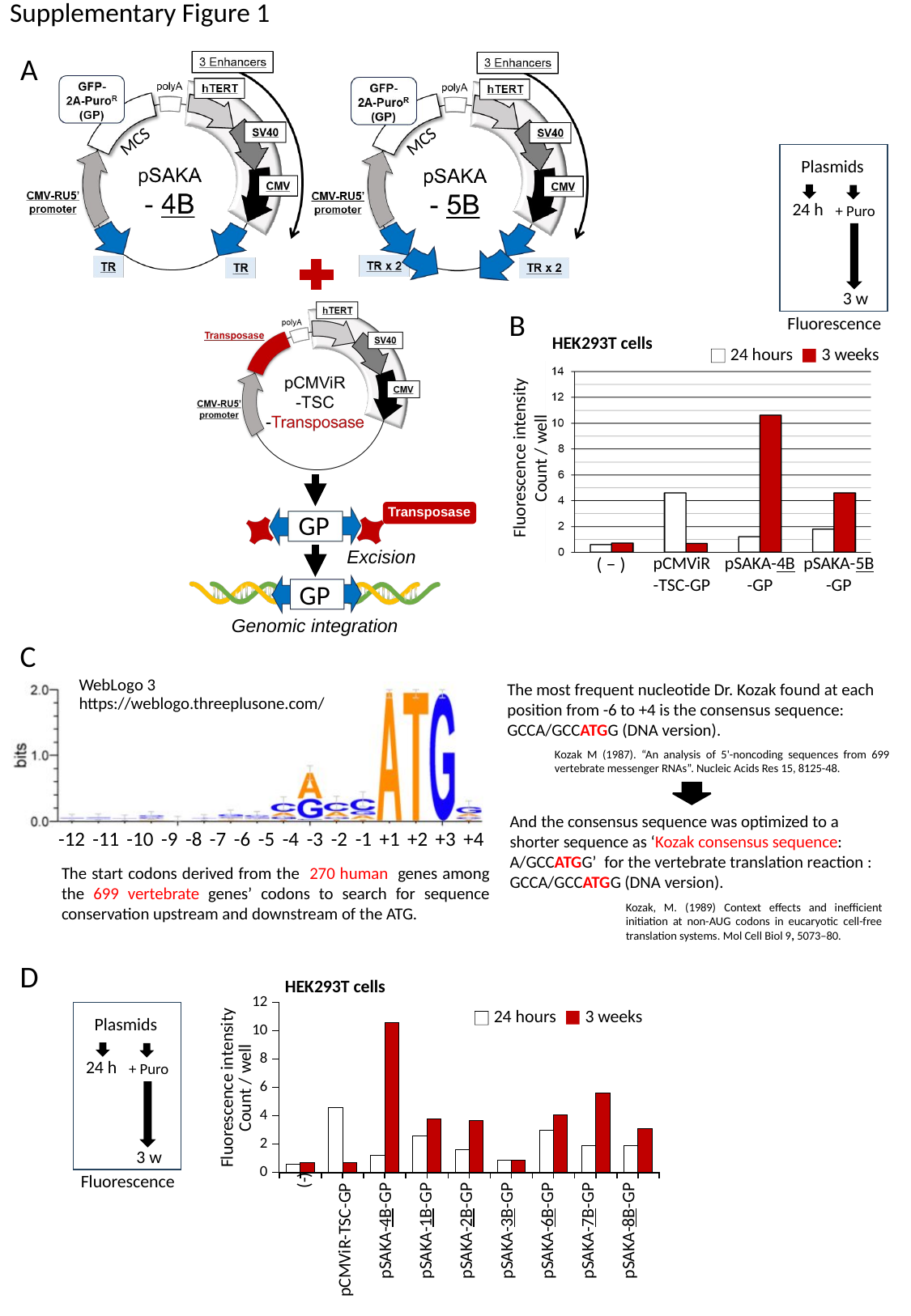

Supplementary Figure 1
A
MCS
MCS
Plasmids
24 h
+ Puro
3 w
B
Fluorescence
HEK293T cells
■ 24 hours ■ 3 weeks
Fluorescence intensity
Count / well
( − )
pCMViR
-TSC-GP
pSAKA-4B
-GP
pSAKA-5B
-GP
Transposase
GP
Excision
GP
Genomic integration
C
WebLogo 3
https://weblogo.threeplusone.com/
-12
-11
-10
-9
-8
-7
-6
-5
-4
-3
-2
-1
+1
+2
+3
+4
The most frequent nucleotide Dr. Kozak found at each position from -6 to +4 is the consensus sequence: GCCA/GCCATGG (DNA version).
Kozak M (1987). “An analysis of 5'-noncoding sequences from 699 vertebrate messenger RNAs”. Nucleic Acids Res 15, 8125-48.
And the consensus sequence was optimized to a shorter sequence as ‘Kozak consensus sequence: A/GCCATGG’ for the vertebrate translation reaction : GCCA/GCCATGG (DNA version).
The start codons derived from the 270 human genes among the 699 vertebrate genes’ codons to search for sequence conservation upstream and downstream of the ATG.
Kozak, M. (1989) Context effects and inefficient initiation at non-AUG codons in eucaryotic cell-free translation systems. Mol Cell Biol 9, 5073–80.
D
HEK293T cells
### Chart
| Category | 24 hours | 3 weeks |
|---|---|---|
| (-) | 0.6 | 0.7 |
| pCMViR-TSC | 4.6 | 0.7 |
| 4G | 1.2 | 10.6 |
| 1G | 2.6 | 3.8 |
| 6G | 1.6 | 3.7 |
| 3G | 0.9 | 0.9 |
| 7G | 3.0 | 4.1 |
| 10G | 1.9 | 5.6 |
| 9G | 1.9 | 3.1 |■ 24 hours ■ 3 weeks
Fluorescence intensity
Count / well
(-)
pSAKA-4B-GP
pSAKA-1B-GP
pSAKA-2B-GP
pSAKA-3B-GP
pSAKA-6B-GP
pSAKA-7B-GP
pSAKA-8B-GP
pCMViR-TSC-GP
Plasmids
24 h
+ Puro
3 w
Fluorescence
